# Case of Necrosis of the Bones of the Head

**Published:** 1865-01

**Authors:** Johnson Gardner

**Affiliations:** Providence, R.I.


Case of Necrosis of the Bones of the Head.—*By Johnson Gardner, M. D., of Providence, R. I.*—About three years since, in 1861, Mrs. ——, of Providence, R. I., now 43 years of age, the mother of nine healthy children, applied to me with chronic laryngitis and ulceration of the tonsils, with the palate partially destroyed and perforated with many large holes.

The whole cavity of the throat was lined with purulent matter, and the disease had extended so far as nearly to destroy the voice, articulation being so imperfectly accomplished that with the greatest apparent effort only now and then could a word be understood. The patient was also suffering with great pain in the head, the integuments being much swollen and inflamed in places over the parietal bones, with one or two open abscesses. In giving the history of her case she began by saying that she had been sick with a violent cough ten or twelve years previous, which continued for a long time, and was so persistent that her friends thought she had consumption; and that Dr. Miller was consulted in her case. That since that time she had employed several other physicians, and although her cough was better, in every other respect her health was much worse. She had become very much emaciated, and presented a pale, cadaverous appearance, with great loss of appetite and strength, and every indication of much suffering; her pulse was about one hundred in a minute.

My first prescription was a solution of thirty grains of nitrate of silver in an ounce of water, which I thoroughly applied to the throat daily with a curved pharyngeal syringe, ending in a perforated bulb; at the time I prescribed twenty drops of tinct. ferri muriat., three times a day. But at the end of a week, finding the result unsatisfactory, that the disease of the throat was not much if at all arrested, and the voice almost gone, I substituted therefore a lotion of acid of nitrate of mercury, and applied it thoroughly, every day or two, in the manner before described. I also gave the patient four grains of iodide of potassium three times a day, and a two-grain pill of sulphate of quinine before each meal. This treatment acted like a charm. The ulceration was gradually lessened, and in the same ratio the voice was restored, till at length the disease of the throat ceased to be troublesome, and no local application, other than a gargle of solution of chlorate of potassa every morning, was required. But as the condition of the throat improved, the pain and swelling of the integuments of the cranium grew gradually worse, and about a year since the suffering had become so great and the discharge from the abscesses so profuse, that I did not believe the patient would long survive. Yet I could think of no better course than to persevere with the internal treatment. Whenever, on probing, I felt that one of the pieces of bone had become loose, I proceeded with the aid of the scalpel and bone forceps to extract it. In this way, one after another, a number of pieces of bone have been removed. The last was taken from the head the 29th of November last, with some difficulty on account of its angular shape and its being entirely covered with integuments. With a single exception near the right eye, all the places from which pieces of bone have been removed are now healed, leaving each a sunken, contracted, indurated cicatrix. The largest specimens are parts of the parietal bones. Two are from the superciliary ridges of the os frontis, one from each, and some of them are from the superior part of the occipital bone. Thus nearly all of the upper part of the head has been invaded by the ravages of this disease. But now, contrary to my long-continued expectations, there is an encouraging improvement in the general health, as well as the local disease. The strength has greatly improved. The voice is entirely restored, and the patient is able to superintend her domestic affairs, and enjoy the pleasures of society.

It may be supposed that the sufferer was a victim of secondary syphilis, but to support this theory no evidence other than what has been narrated can be found. The patient is a respectable woman, and there appears no good reason why her veracity should be questioned. She positively asserts that she never had a vestige of this disease in any form, and that neither her husband or any other member of her family ever had it, to her knowledge. Besides her children are, and have been perfectly healthy.—*Boston Med. and Surg. Jour.*

